# Effect of a 12-Week Concurrent Training Intervention on Cardiometabolic Health in Obese Men: A Pilot Study

**DOI:** 10.3389/fphys.2021.630831

**Published:** 2021-02-11

**Authors:** Francisco J. Amaro-Gahete, Jesús G. Ponce-González, Juan Corral-Pérez, Daniel Velázquez-Díaz, Carl J. Lavie, David Jiménez-Pavón

**Affiliations:** ^1^EFFECTS-262 Research group, Department of Physiology, Faculty of Medicine, University of Granada, Granada, Spain; ^2^PROmoting FITness and Health Through Physical Activity Research Group, Department of Physical Education and Sports, Faculty of Sport Sciences, University of Granada, Granada, Spain; ^3^MOVE-IT Research Group, Department of Physical Education, Faculty of Education Sciences, University of Cádiz, Cádiz, Spain; ^4^Biomedical Research and Innovation Institute of Cádiz Research Unit, Puerta del Mar University Hospital, University of Cádiz, Cádiz, Spain; ^5^Department of Cardiovascular Diseases, John Ochsner Heart and Vascular Institute, Ochsner Clinical School, The University of Queensland School of Medicine, New Orleans, LA, United States

**Keywords:** concurrent training, cardiac function, obesity, body composition, energy metabolism

## Abstract

The present study aimed to investigate the effects of a 12-week concurrent training intervention on cardiometabolic health in obese men. Twelve obese men (42.5 ± 5.3 years old) participated in the current 12−week randomized controlled trial with a parallel group design. The participants were randomly assigned to a concurrent training group or to a no-exercise control group. Anthropometry and body composition assessment were determined by electrical bio-impedance. Blood samples were obtained and a cardiometabolic risk Z-Score was calculated. Energy metabolism-related parameters [i.e., resting metabolic rate (RMR), respiratory quotient (RQ), and substrate oxidation in both resting conditions and during exercise] were determined by indirect calorimetry. Echocardiographic studies were performed using an ultrasound system equipped with a transducer to measure cardiac function. A significant decrease of weight (Δ = −4.21 kg; i.e., primary outcome), body mass index (Δ = −1.32 kg/m^2^), fat mass (FM; Δ = −3.27 kg), blood pressure (BP; Δ = −10.81 mmHg), and cardiometabolic risk Z-Score (Δ = −0.39) was observed in the exercise group compared with the control group (all *P* < 0.05), while no significant changes were noted in waist circumference (WC), lean mass (LM), bone mineral content, glycemic and lipid profiles, liver function, nor in energy metabolism-related parameters (all *P* > 0.1). Moreover, a significant increment of left ventricular (LV) end diastolic diameter (Δ = −4.35 mm) was observed in the exercise group compared with the control group (*P* = 0.02). A 12-week concurrent training intervention is an effective strategy to induce weight and fat loss with simultaneous reductions of BP and cardiometabolic risk, and improving cardiac function in obese men.

## Introduction

Globally, obesity has reached epidemic proportions in the current 21st century and is associated with higher risk of premature mortality (Lavie et al., 2018b). Indeed, obesity is an independent risk factor for cardiovascular (CV) disease (CVD, including hypertension, coronary heart disease, heart failure, and sudden cardiac death) (Go et al., 2014; Ng et al., 2014; Jaacks et al., 2019; Jiménez-Pavón et al., 2019), and has been connected with several comorbidities, including dyslipidemia, insulin resistance, diabetes mellitus (DM), low metabolic flexibility, or left ventricular (LV) hypertrophy, among others (Perumareddi, 2019). Health care spending derived from obesity-related diseases has exponentially increased during the last decade and is expected to continue rising (Lavie et al., 2018a). Therefore, the application of cost-effective measures to reduce obesity and its related health burden are of clinical and scientific interest.

Over the past years, different strategies have been found to improve cardiometabolic health in individuals with obesity. Physical activity (PA) is considered an integral approach for obese individuals, not only for weight loss goals but also for reducing the risk of CVD, type 2 DM, and all-cause mortality (Petridou et al., 2018). World Health Organization has recently updated a consensus statement regarding the global recommendations on PA for health promotion (i.e., 150 or 75 min per week of moderate or vigorous intensity aerobic PA/exercise, respectively, plus resistance exercise twice per week; World Health Organization [WHO], 2015; Piercy et al., 2018). The combination of aerobic and resistance training (i.e., concurrent training) has been positioned as a promising tool to improve CV and metabolic profiles in both healthy individuals (Bennie et al., 2018) and patients with cardiometabolic diseases (Álvarez et al., 2019). Concretely, previous studies have reported that concurrent training is an effective antihypertensive (Corso et al., 2016) and anti-inflammatory therapy (Libardi et al., 2012), improving in turn the glycemic and lipid profiles (Braga de Mello et al., 2019) as well as hepatic function (Monteiro et al., 2015). Nevertheless, these previous studies included individuals with different biological characteristics making it necessary for further investigations attaining patients with cardiometabolic disturbances.

To the best of our knowledge, there is a lack of studies in obese persons investigating not only the effects of concurrent training on body composition and cardiometabolic profile, but also on liver function, energy metabolism, or CV function, all of them involved in further obesity-related complications. Concretely, metabolic flexibility (i.e., the ability to respond or adapt to conditional changes in metabolic demand) has been propagated to explain insulin resistance and mechanisms governing fuel selection between glucose and fatty acids, highlighting that patients with obesity and type 2 diabetes suffer from metabolic inflexibility. Similarly, liver function alterations such as non-alcoholic fatty liver disease **are** usually present in patients with metabolic syndrome and central abdominal obesity (Milić et al., 2014). Considering obesity as a multifactorial disease, it would be of clinical interest to understand the specific effects of concurrent training on those parameters which are altered in obese persons. Therefore, the present study aimed to investigate the effects of a 12-week concurrent training intervention on cardiometabolic health (i.e., body composition, glycemic and lipid profiles, liver function, energy metabolism, and cardiac function) in obese men.

## Materials and Methods

### Research Design and Subjects

A 12-week intervention study with a parallel-group design was conducted following the Consolidated Standards of Reporting Trials guidelines (Welch et al., 2017). After the baseline assessment, participants were randomly assigned into two different groups using computer-generated simple randomization: (i) the control group (no exercise—maintaining their habitual lifestyle) and (ii) the concurrent training group. The participant’ allocation was blinded to the assessment staff. Participants were instructed to maintain their dietary and PA habits. The same exercise intervention was offered to the participants of the control group after completing the intervention.

Participants were obese sedentary men (35–55 years) with no comorbidities. The experimental design and study protocols were conducted strictly following the last revised ethical guidelines of the Declaration of Helsinki. The current pilot study was approved by the Ethics Committee on Human Research at the University of Leon and all participants signed an informed consent. The participants were recruited from the province of Cadiz (Spain) using social networks and local media. Inclusion criteria were: (i) to have a BMI > 30 kg/m^2^; (ii) to be sedentary [less than 150 min/week of moderate-intensity PA (i.e., self-reported) over the last 6 months]; (iii) to present a stable weight over the last 12 weeks; (iv) to be free of any chronic disease that could be aggravated by exercise training; and (v) not to consume any chronic mediation (i.e., self-reported) over the last 6 months. Baseline and follow-up assessment were performed at the same setting (Physical Activity and Exercise physiology Laboratory at the Faculty of Education Sciences, University of Cádiz).

### Concurrent Training Intervention

The participants included in the concurrent training group performed a 12-week intervention based on the updated PA recommendations provided by the World Health Organization (World Health Organization [WHO], 2015). All sessions were conducted under the supervision of an accredited exercise physiologist. A training frequency of three sessions/week was selected. Each training session lasted 60 min and consisted of a combination of aerobic and resistance exercises. Aerobic training intensity was fixed at 60–70% of the heart rate reserve, while resistance training intensity was set at 6–7 of their subjective rates of perceived exertion. The participants were instructed to complete three to four sets which included a total of six to eight aerobic and resistance exercises following a circuit training methodology. The rest between sets was 60–120 s. Treadmill and cycle-ergometer were used to complete the aerobic training, whereas weight bearing and free-weights exercises (i.e., using both dumbbells and bars) were used to perform the resistance training involving the main upper and lower body muscle groups (i.e., lateral pull down, dips, deadlift, squat, or bench press among others). A dynamic standardized warm-up was performed before the beginning of the main part based on mobility and activation exercises, and a cooling-down protocol (i.e., stretching exercises) was conducted at the end of the training session. Exercise’ intensity was continuously monitored during all sessions using a Polar team 2 system (Polar Team 2 system, Polar Electro Oy, Kempele, Finland). No adverse events were observed.

### Procedures

The baseline and post-intervention measurement were organized on 2 days: (i) day 1: medical examination and fasting blood determinations and (ii) day 2: anthropometry and body composition, blood pressure (BP), energy metabolism-related parameters, cardiorespiratory fitness (CRF), and echocardiography. We also used accelerometers to objectively measure PA and we controlled the dietary intake by three 24 h recalls.

#### Anthropometry and Body Composition

Anthropometry and body composition assessments were conducted before and after the intervention program. Weight (primary outcome) and height were determined using a validated scale and stadiometer (SECA 225, Hamburg, Germany) without shoes and with light clothing. Subsequently, the BMI was calculated as weight divided by height^2^. Waist circumference (WC) was measured at the mid-point between the bottom of the rib cage and the iliac crest at the end of a normal expiration.

Electrical bio-impedance (TANITA-MC780MA, Barcelona, Spain) was used to estimate fat mass (FM), lean mass (LM), and bone mineral content (g) following the manufacturer’s recommendations.

#### Blood Samples

Venous blood samples were obtained from the antecubital vein and collected in ethylenediamine tetra-acetic acid-containing tubes in fasting conditions. All samples were centrifuged at 4000 r/min for 10 min at 4°C, and subsequently stored at −80°C until further analysis. Plasma glucose, insulin, total cholesterol, high-density lipoprotein cholesterol (HDL-C), triglycerides (TGs), glutamic oxaloacetic transaminase (GOT), glutamic-pyruvic transaminase (GPT), γ-glutamyl transferase (γ-GT), C-reactive protein (CRP), and leptin were determined using conventional methods (i.e., spectrophotometry, chemiluminescence assay, and enzyme-linked immunosorbent assay). The homeostatic model assessment of insulin resistance (HOMA-IR) index was then calculated as (*plasma insulin*) × *plasma glucose*/22.5 (Ascaso et al., 2001). Low-density lipoprotein cholesterol (LDL-C) was determined as (*total cholesterol*) – (*HDL-C*) – 0.45 × (*TGs*). Fatty liver index was also calculated using a previously validated equation (Bedogni et al., 2006):

F⁢a⁢t⁢t⁢y⁢L⁢i⁢v⁢e⁢r⁢I⁢n⁢d⁢e⁢x⁢(F⁢L⁢I)=

$$(e^{0.953*loge\left(TGs\right)+0.139*BMI+0.718*loge\left(\gamma -\text{GT}\right)+0.053*\mathrm{WC}-15.745)})*100$$

A cardiometabolic risk Z-score was determined considering the clinical parameters proposed by the International Diabetes Federation to diagnose metabolic syndrome (Carracher et al., 2018) (i.e., WC, BP, plasma glucose, HDL-C, and TGs). These outcomes were standardized as (*value – mean*)/*standard deviation.* HDL-C standardized value was multiplied by −1 since we aimed to reflect a high cardiometabolic risk with higher values. The mean of the five standardized values was considered the cardiometabolic risk Z-score obtaining a standard deviation of 1 and a mean of 0 by definition.

#### BP

Participants were sitting in a chair, relaxed with their feet firmly on the floor. After 5 min, systolic and diastolic BPs were assessed using an automatic monitor Omron M3 intelligence advice (HEM-7051-E, Kyoto, Japan), which has been previously validated, on the non-dominant arm following the recommendations of the European Heart Society (Whelton and Williams, 2018). A minimum of three measurements were taken 1 min apart, and the mean value was subsequently calculated as:

$$\mathrm{Mean}\,\mathrm{arterial}\,\mathrm{BP}\,\left(\text{MAP}\right)\,=\frac{S\,y\,s\,t\,o\,l\,i\,c\,B\,P+\left(2*D\,y\,a\,s\,t\,o\,l\,i\,c\,B\,P\right)}{3}$$

#### Energy Metabolism

Resting metabolic rate (RMR) was assessed in the morning after an overnight fast. We instructed the participants to avoid any exertion after waking up the testing day, to refrain from any moderate or vigorous PA before the testing (i.e., 24 and 48 h, respectively), to sleep as usual, to maintain their usual diet and to avoid the intake of alcohol and caffeine the day before. The environmental conditions were strictly controlled (temperature 20–22°C, humidity 60–65%). After their lab arrival, the participants were instructed to lie on a comfortable bed in a supine position for 5 min before the beginning of the RMR test that lasted 30 min (Fullmer et al., 2015). Oxygen consumption and carbon dioxide production were obtained by indirect calorimetry methods using a metabolic cart Jaeger MasterScreen CPX^®^ (CareFusion, San Diego, CA, United States) which was previously calibrated following the manufacturer’s guidelines (i.e., gas and volume calibrations). The participants were asked not to sleep, talk, or fidget, and to breath normally. For the calculation of the RMR, we averaged the ventilatory parameters every 20 s. The first 10 min was discarded (Fullmer et al., 2015), and we calculated the coefficients of variance (CV) for oxygen consumption, carbon dioxide production, respiratory quotient (RQ), and minute ventilation every 5 min period (Fullmer et al., 2015; Sanchez-Delgado et al., 2018). Then, the periods that met the steady-state criteria for the ventilatory parameters (i.e., CV < 10% for oxygen consumption, CV < 10% for carbon dioxide production, CV < 5% for RQ, and CV < 10% for minute ventilation) were chosen, considering the period with the lowest average CV for these ventilatory outcomes for further analysis (Fullmer et al., 2015; Sanchez-Delgado et al., 2018). RMR and substrates oxidation (i.e., fat and carbohydrate oxidation) were determined through the stoichiometry equations of Weir and Frayn, respectively.

Maximal fat oxidation (MFO) and the intensity that elicits MFO (Fat_max_) were determined through a graded exercise test on cycloergometer (Lode Excalibur, Groningen, Netherlands). This test consisted on cycling at 15 W keeping a constant cadence of 60–80 r/min for 3 min increasing the workload 15 W every step until reaching an RQ of 1.0 (Tsujimoto et al., 2011). Oxygen consumption and carbon dioxide production were determined by indirect calorimetry during the exercise protocol, using a metabolic cart Jaeger MasterScreen CPX^®^ (CareFusion, San Diego, CA, United States), previously calibrated as explained above, and employing a face mask equipped with a metabolic flow sensor (CareFusion, San Diego, CA, United States) for gas data collection. We averaged the ventilatory parameters every 20 s, and fat oxidation values were estimated considering oxygen consumption and carbon dioxide production values averaged over the final 1 min of each 3-min stage (Amaro-Gahete et al., 2019c), using the Frayn stoichiometric equation and considering the urinary nitrogen excretion as negligible. MFO and Fat_max_ were calculated using a third polynomial regression curve with an intersection at 0;0, plotting fat oxidation values obtained in each period of the graded exercise test against the relative exercise intensity (Amaro-Gahete et al., 2019c).

#### Dietary Intake

Dietary intake was determined by a qualified and trained researcher on diet assessments through three non-consecutive 24 h recalls (including one weekend day). Food consumption was obtained by the DIAL^®^ software for Windows, version 3.7.1.0. Subsequently, food consumption was transformed into energy and macronutrient intakes.

#### PA and Sedentary Behavior (SB)

Levels of PA and sedentary behavior (SB) were measured with a hip-worn ActiGraph GT3X + accelerometer (ActiGraph, Pensacola, FL, United States). The participants were asked to wear the accelerometer for seven consecutive days during the 24 h. After data collection, the ActiLife v.6.2.2 software (ActiGraph, Pensacola, FL, United States) was used to their processing, excluding those participants that did not wear the accelerometer for at least 16 h/day during at least 4 days (including at least one weekend day).

#### CRF

Maximum oxygen uptake (VO_2max_) was determined just after the MFO and Fat_max_ determination. After a short break (≈3 min), the second phase of the graded exercise test was initiated starting with the last step’ intensity of the previous phase and increasing the load 15 W each minute. The participants were asked to maintain a constant cadence of 60–80 r/min until they reached voluntary exhaustion. Oxygen consumption and carbon dioxide production were also obtained via indirect calorimetry, gathering data as for MFO and Fat_max_ testing (see above). The criteria for achieving VO_2_max were: (i) to attain an RQ higher than 1.1, (ii) to reach a plateau in oxygen consumption (change lower than 100 mL/min in the last 30-s stages), and (iii) to show a heart rate between 10 beats/min of the age-predicted maximal heart rate (Midgley et al., 2007; Amaro-Gahete et al., 2019a). We considered the peak oxygen uptake value during the exercise test when these criteria were not met (Midgley et al., 2007; Amaro-Gahete et al., 2019a).

#### Echocardiography

Echocardiographic studies were performed by single experienced cardiologist (blinded to the participants’ assignment group) using an ultrasound system (Sonosite-Edge, Amsterdam, Netherlands) equipped with a transducer. Cardiac mass, volumes, and dimensions were measured according to the current recommendations. Mitral inflow velocities were determined using pulsed-wave Doppler recording velocities end-expiration. LV diastolic function was measured following the EAE/ASE consensus guidelines (Nagueh et al., 2008) obtaining E wave, A wave, E/A ratio, and E wave deceleration time.

### Statistical Analysis

Descriptive and exploratory analyses of all the study outcomes were conducted to check statistical assumptions, distributions, and imbalances between the study groups. Student’s *t*-tests for unpaired values were applied to determine intergroup differences (i.e., control vs. intervention group) at the baseline in the study’ outcomes. The intervention effects on primary and secondary parameters were assessed through linear mixed-effects models considering individual measures of growth as the function of randomly assigned group, time, and its interaction. We conducted these estimations using the restricted maximum-likelihood method which includes an unstructured covariance matrix to adjust for within-participant clustering resulting from the repeated-measures design. We adjusted the model for the baseline values of each outcome analyzed. Lastly, we also calculated the standardized effect sizes using Cohen’s d coefficients. The Statistical Package for the Social Sciences v.22.0, (IBM Corporation, Chicago, IL, United States) was used to perform the analyses.

## Results

Twelve obese sedentary men (mean age = 42.5 years) were participated in this trial. Participants attended to ≥ 86% (31 of 36 sessions) of their supervised exercise from baseline to week 12 and they showed a percentage of compliance of ∼90% for exercise’ intensity and 100% for exercise’ volume. There were no significant differences between groups in any variable at the baseline (all *P* ≥ 0.09).

A significant decrease of weight, BMI, and FM was observed in the concurrent training group compared with the control group (all *P* < 0.049; Table 1), while no significant changes were noted in height, WC, LM, and bone mineral content (all *P* > 0.1; Table 1).

**TABLE 1:** Changes in cardiometabolic health outcomes after 12-week intervention among control and concurrent training group.

	Control group (*n* = 6)			Concurrent training group (*n* = 6)			Net effect	
			
	Baseline Mean (SD)	After 12 weeks Mean (SD)	Δ (SE)	Baseline Mean (SD)	After 12 weeks Mean (SD)	Δ (SE)	Mean difference (95% CI)	Standardized mean difference (95% CI)
Age (years)	43.7 (6.1)			41.3 (4.4)				
**Anthropometry and body composition**								
Weight (kg)	101.4 (12.9)	103.5 (14.4)	2.13 (0.88)	97.5 (15.4)	95.4 (12.9)	−2.08(1.33)	4.21(0.45,8.37)	1.23(0.01,2.45)*
Body mass index (kg/m^2^)	32.5 (3.0)	33.2 (3.3)	0.65 (0.26)	32.1 (3.6)	31.4 (2.7)	−0.67(0.42)	1.32(0.01,2.63)	1.23(0.01,2.45)*
Waist circumference (cm)	108.0 (6.1)	107.9 (8.0)	−0.05(1.52)	105.4 (9.4)	104.1 (8.3)	−1.23(0.98)	1.18(−2.79,5.15)	0.46(−1.08,1.99)
Fat mass (kg)	28.5 (7.3)	30.7 (7.6)	2.15 (0.70)	27.8 (6.6)	26.6 (4.8)	−1.12(0.83)	3.27(0.56,5.97)	1.36(0.23,2.48)*
Lean mass (kg)	69.3 (6.9)	69.3 (7.4)	0.00 (0.71)	66.3 (9.3)	65.3 (8.4)	−0.93(0.51)	0.93(−0.69,2.56)	0.82(−0.61,2.25)
Bone mineral content (kg)	3.60 (0.32)	3.58 (0.36)	−0.03(0.03)	3.43 (0.46)	3.40 (0.41)	−0.03(0.33)	0.01(−0.98,0.11)	0.12(−1.45,1.70)
**Blood pressure**								
Systolic blood pressure (mm Hg)	131.5 (16.9)	130.0 (18.6)	−1.50(1.21)	131.4 (22.0)	120.2 (14.8)	−11.22(3.63)	9.72(0.38,19.07)	1.15(0.04,2.26)*
Diastolic blood pressure (mm Hg)	83.2 (6.3)	86.9 (8.1)	3.75 (1.80)	86.6 (13.9)	79.0 (8.8)	−7.61(3.40)	11.36(1.02,21.70)	1.29(0.12,2.47)*
Mean blood pressure (mm Hg)	99.3 (9.2)	101.3 (11.4)	2.00 (1.58)	101.5 (16.5)	92.7 (10.4)	−8.81(3.45)	10.81(1.79,19.84)	1.26(0.21,2.30)*
**Glycemic profile**								
Plasma glucose (mg/dL)	101.5 (3.9)	100.3 (3.8)	−1.25(2.21)	94.8 (9.1)	93.8 (8.5)	−1.00(1.52)	−0.25(−6.39,5.89)	−0.07(−1.76,1.63)
Plasma insulin (UI/mL)	13.9 (4.2)	19.6 (11.1)	5.71 (4.00)	8.6 (2.0)	8.3 (3.8)	−0.26(2.38)	5.97(−4.50,16.44)	0.86(−0.65,2.37)
HOMA-IR	3.47 (1.02)	4.84 (2.75)	1.37 (1.05)	2.00 (0.52)	1.96 (0.98)	−0.04(0.55)	1.42(−1.22,4.05)	0.82(−0.71,2.35)
**Lipid profile**								
Total cholesterol (mg/dL)	216.0 (59.8)	212.5 (47.2)	−3.50(15.09)	198.0 (22.6)	211.8 (42.5)	13.80 (13.4)	−17.30(−65.02,30.42)	0.58(−2.19,1.03)
HDL-C (mg/dL)	48.8 (9.6)	46.8 (1.7)	−2.00(5.35)	40.8 (5.0)	43.8 (4.9)	3.00 (0.45)	−5.00(−16.18,6.18)	−0.70(−2.28,0.87)
LDL-C (mg/dL)	145.0 (50.3)	142.3 (41.5)	−2.75(9.29)	130.8 (22.0)	147.6 (37.7)	16.80 (9.07)	−19.55(−50.60,11.50)	−0.93(−2.41,0.55)
Triglycerides (mg/dL)	112.0 (53.9)	118.5 (44.6)	6.50 (16.98)	131.4 (70.5)	102.0 (56.5)	−29.40(30.55)	35.90(−53.28,125.08)	0.64(−0.95,2.24)
**Liver function**								
GOT (IU/L)	29.3 (8.5)	33.0 (2.8)	3.75 (2.93)	21.6 (8.1)	21.2 (7.6)	−0.40(0.68)	4.15(−2.19,10.49)	0.96(−0.51,2.42)
GPT (IU/L)	44.0 (27.5)	46.5 (21.6)	2.50 (3.12)	22.0 (7.2)	23.8 (13.4)	1.80 (3.76)	0.70(−11.29,12.69)	0.10(−1.59,1.79)
γ-GT (IU/L)	68.5 (75.2)	73.3 (62.7)	4.75 (11.28)	27.2 (8.23)	27.2 (13.3)	0.00 (3.56)	4.75(−20.55,30.05)	0.31(−1.36,1.99)
Fatty liver index	90.8 (6.9)	93.6 (5.0)	2.77 (8.40)	67.9 (62.2)	62.2 (16.5)	−5.64(7.39)	8.42(−25.51,42.34)	0.63(−1.91,3.17)
Cardiovascular risk Z-Score	0.023 (0.525)	0.203 (0.416)	0.180 (0.089)	0.084 (0.506)	−0.136(0.358)	−0.220(0.148)	0.39(0.04,0.83)	1.30(0.12,2.51)*
**Other biochemical parameters**								
Protein C reactive (mg/dL)	11.7 (18.1)	8.2 (9.4)	−3.53(4.47)	1.6 (0.8)	1.8 (1.0)	0.18 (0.17)	−3.71(−13.01,5.60)	−0.64(−2.23,0.96)
Leptin (ng/mL)	19.8 (5.1)	21.0 (6.4)	1.18 (1.00)	23.3 (12.1)	15.7 (3.0)	−7.58(5.03)	8.76(−4.89,22.40)	0.94(−0.52,2.41)
**Energy metabolism**								
RMR (kcal/kg/day)	18.78 (1.00)	20.24 (1.75)	1.46 (1.72)	20.13 (2.53)	20.55 (1.77)	0.41 (1.98)	−1.09(−2.02,4.19)	0.57(−0.59,1.72)
RQ	0.867 (0.172)	0.861 (0.173)	−0.006(0.021)	0.831 (0.194)	0.824 (0.188)	−0.007(0.028)	−0.001(−0.018,0.014)	−0.04(−1.17,1.09)
RFox (g/min)	0.084 (0.026)	0.102 (0.044)	0.018 (0.014)	0.081 (0.027)	0.082 (0.023)	0.001 (0.008)	0.02(−0.02,0.05)	0.71(−0.76,2.18)
RFox (% RMR)	50.8 (19.3)	60.6 (25.8)	9.76 (11.95)	51.5 (16.6)	50.7 (12.5)	−0.75(4.92)	10.50(−25.27,46.26)	0.61(−1.46,2.67)
RCHox (g/min)	0.148 (0.065)	0.131 (0.086)	−0.017(0.045)	0.154 (0.049)	0.153 (0.035)	−0.001(0.017)	−0.02(−0.15,0.12)	−0.27(−2.48,1.95)
RCHox (% RMR)	46.4 (17.3)	38.8 (25.4)	−7.66(10.98)	49.1 (17.2)	48.0 (12.5)	−1.12(4.95)	−6.53(−39.24,26.18)	−0.41(−2.46,1.64)
MFO (g/min)	0.24 (0.03)	0.26 (0.07)	0.019 (0.037)	0.24 (0.05)	0.25 (0.03)	0.010 (0.020)	0.01(−0.08,0.10)	0.17(−1.39,1.75)
Fat_max_ (% VO_2_max)	48.8 (9.5)	54.6 (6.9)	5.85 (3.91)	51.5 (6.8)	53.1 (7.3)	1.58 (3.75)	4.28(−8.67,17.24)	0.50(−1.02,2.03)
**Dietary intake**								
Energy intake (kcal/day)	2868 (972)	2796 (680)	−71.5(370.9)	2911 (518)	2436 (408)	−474.7(272.7)	403.2(−633.6,1439.9)	0.59(−0.91,2.09)
Carbohydrate intake (g/day)	281.0 (81.1)	277.8 (121.7)	−3.25(47.53)	238.3 (47.9)	284.0 (42.6)	45.67 (30.27)	48.92(−74.04,171.88)	0.60(−0.90,2.10)
Sugar intake (g/day)	114.6 (32.6)	84.7 (22.4)	−29.83(21.31)	111.1 (20.0)	91.5 (19.1)	−19.63(8.91)	−10.19(−73.91,53.53)	−0.34(−2.47,1.79)
Far intake (g/day)	103.3 (56.1)	116.2 (37.6)	2.93 (25.34)	126.7 (23.8)	104.3 (26.7)	−22.35(15.95)	25.28(−39.91,90.46)	0.58(−0.92,2.09)
Protein intake (g/day)	130.2 (39.4)	121.6 (38.4)	−8.55(14.33)	110.5 (23.4)	107.5 (15.2)	−2.97(8.96)	−5.58(−48.26,37.09)	−0.24(−1.80,1.33)
Alcohol intake (g/day)	13.4 (11.6)	14.1 (13.5)	−0.70(6.02)	19.3 (23.8)	10.9 (7.6)	−8.40(10.13)	9.10(−22.11,40.31)	0.45(−1.09,1.98)
**Physical activity and sedentary behavior**								
Sedentary time (min/day)	7122.5 (1694.2)	6834.3 (1018.9)	−288.25(754.93)	7323.4 (478.5)	7291.2 (855.9)	−32.20(205.50)	−256.1(−1917.9,1405.8)	−0.26(−1.94,1.42)
LPA (min/day)	9807.0 (268.9)	9782.5 (89.2)	−24.50(147.99)	9864.8 (118.6)	9833.4 (47.3)	−31.40(66.08)	6.90(−347.87,361.67)	0.03(−1.66,1.73)
MPA (min/day)	272.8 (268.9)	296.3 (88.8)	23.50 (147.81)	188.8 (92.9)	239.4 (43.6)	50.60 (45.83)	−27.10(−357.76,303.56)	−0.14(−1.83,1.55)
VPA (min/day)	0.3 (0.5)	0.3 (0.5)	0.00 (0.41)	3.4 (3.1)	25.8 (57.1)	22.40 (25.94)	22.40(−47.16,91.96)	0.52(−1.10,2.15)
MVPA (min/day)	273.0 (268.9)	296.5 (89.2)	23.50 (147.99)	215.2 (118.6)	145.6 (47.27)	30.40 (66.08)	−6.90(−361.67,347.87)	−0.03(−1.73,1.66)
**Cardiorespiratory fitness**								
VO_2_max (mL/min)	2596.3 (472.2)	2876.0 (524.4)	279.75 (95.79)	2620.3 (573.3)	2913.5 (561.4)	293.17 (299.34)	−13.42(−893.75,866.92)	−0.24(−1.60,1.55)
VO_2_max (mL/kg/min)	25.6 (2.3)	27.7 (2.6)	2.19 (0.88)	27.4 (7.4)	30.7 (5.5)	3.31 (2.97)	−1.11(−8.74,6.51)	−0.20(−1.57,1.17)
**Echocardiography**								
Cardiac mass (g)	207.4 (32.0)	181.8 (42.3)	−25.57(7.08)	192.6 (27.8)	197.5 (21.9)	4.88 (15.3)	−30.45(−72.41,11.51)	−1.01(−2.39,0.38)
Ejection fraction (%)	65.8 (6.2)	61.0 (1.8)	−4.75(2.69)	66.2 (5.7)	61.6 (7.4)	−4.60(3.87)	−0.15(−11.93,11.63)	−0.02(−1.72,1.67)
LV end diastolic diameter (mm)	52.5 (5.2)	50.8 (3.5)	−1.75(1.25)	52.6 (3.2)	55.2 (0.4)	2.60 (1.54)	−4.35(−9.22,−0.52)	−1.28(−2.50,−0.14)*
LV end systolic diameter (mm)	26.3 (4.0)	30.5 (2.6)	4.25 (1.89)	33.4 (3.6)	34.2 (3.7)	0.80 (0.80)	3.45(−2.20,9.10)	1.08(0.69,2.84)
LV end systolic volume (mL)	21.5 (10.3)	28.0 (15.3)	6.50 (5.33)	22.4 (2.4)	34.6 (10.6)	12.20 (4.62)	−5.70(−22.31,10.91)	−0.56(−2.18,1.06)
E wave (cm/s)	87.3 (12.5)	81.8 (23.7)	−5.50(8.93)	70.2 (20.8)	67.6 (16.3)	−2.60(6.17)	−2.90(−27.75,21.95)	−0.20(−1.88,1.49)
A wave (cm/s)	55.8 (3.7)	59.8 (2.6)	4.00 (3.16)	65.8 (17.4)	61.8 (8.4)	−4.00(8.61)	8.00(−15.51,31.51)	−0.55(−1.08,2.18)
E/A	1.57 (0.26)	1.37 (0.38)	−0.20(0.08)	1.07 (0.13)	1.10 (0.26)	0.04 (0.16)	0.24(−0.69,0.21)	0.81(−2.34,0.71)
E wave deceleration time (ms)	205.0 (20.4)	212.5 (24.7)	7.50 (21.26)	261.0 (43.8)	223.0 (31.3)	−38.00(25.62)	45.50(−36.17,127.17)	0.85(−0.67,2.36)

Abbreviations: SD, standard deviation; SE, standard error; CI; confidence interval; HOMA, homeostasis model assessment; HDL-C, high-density lipoprotein cholesterol; LDL-C, low-density lipoprotein cholesterol; GOT, Glutamic oxaloacetic transaminase; GPT, glutamic-pyruvic transaminase; γ-GT, γ-glutamyl transferase; RMR, resting metabolic rate; RFox, resting fat oxidation; RCHox, resting carbohydrate oxidation; MFO, maximal fat oxidation during exercise; Fatmax, intensity that elicits MFO; LPA, physical activity levels at light intensity; MPA, physical activity levels at moderate intensity; VPA, physical activity levels at vigorous intensity; MVPA, physical activity levels at moderate-vigorous intensity; VO2max, maximal oxygen uptake; LV, left ventricle. Analyses were performed using multilevel mixed analysis including group as a fixed variable (control group vs. exercise).

*Significant differences between groups (*P* < 0.05).

Multi-level mixed analyses, adjusting for baseline values, revealed a significant reduction of BP (i.e., systolic, diastolic, and MAP) and CV risk Z-Score in the concurrent training group compared with the control group (all *P* < 0.044; Table 1), whereas no significant differences were seen between groups with respect to the change in both glycemic (i.e., plasma glucose, plasma insulin, and HOMA-IR) and lipid profiles (i.e., total cholesterol, HDL-C, LDL-C, and TGs), as well as in liver function (i.e., GOT, GPT, γ-GT, and FLI) and other biochemical parameters, such as CRP and leptin (all *P* > 0.17; Table 1).

There were no differences between groups neither in energy metabolism-related parameters (i.e., RMR, RQ, resting substrates oxidation, MFO, and Fat_max_), dietary intake (i.e., energy, macronutrients, and alcohol intake) and PA levels, and sedentary time nor in VO_2max_ (all *P* > 0.2; Table 1).

We observed a significant increment of LV end diastolic diameter in the concurrent training group compared with the control group (*P* = 0.02; Table 1), while no significant differences were noted in cardiac mass, ejection fraction, LV end systolic diameter, LV end systolic volume, E wave, A wave, E/A, and E wave deceleration time (all *P* > 0.2; Table 1).

## Discussion

The current study sought to elucidate whether a 12-week concurrent training intervention improves cardiometabolic health in obese men. As we expected, the main findings of the present work were that compared to the control group, the participants included in the exercise group benefited from a significant improvement in weight management, FM loss, BP, cardiometabolic risk, and cardiac function, while no significant changes were noted in neither liver function nor energy metabolism-related parameters.

Previous studies have reported that concurrent training is an efficient tool to reduce weight and FM while increasing LM (Ferreira et al., 2010; Michell et al., 2014; Amaro-Gahete et al., 2019). Michell et al. (2014) showed an increment of LM and a decrement of FM in response to 24-week concurrent training intervention which consisted of three 40-min sessions/week combining aerobic training (i.e., 55–70% of maximum oxygen uptake intensity) and resistance training (65–85% of one maximum repetition intensity) in sedentary men. Ferreira et al. (2010) found that a 10-week concurrent intervention characterized by three 60-min sessions/week of aerobic and resistance training at moderate intensity also induces FM loss and LM gain in sedentary women. Similarly, a recent study conducted in our laboratory (Amaro-Gahete et al., 2019) revealed a significant decrease of FM and an increase of LM after a 12-week concurrent training intervention based on the minimum PA recommended by the World Health Organization (World Health Organization [WHO], 2015; Piercy et al., 2018) in middle-aged sedentary adults. These results partially concur with those obtained in the current study, since we also observed a significant decrease of both weight and FM in the current cohort. However, we did not observe significant changes in LM after the exercise intervention compared with the control group. The main reasons that could explain this discrepancy among studies are the different duration of the above-mentioned concurrent training intervention (i.e., ranged from 10 to 24 weeks) and the different initial weight status of the participants (i.e., normal-weight vs. obese), which could imply different metabolic, hormonal, and molecular responses to a similar exercise stimulus.

Concurrent training has also been proposed as an excellent method to improve cardiometabolic health through the management of glycemic and lipid profile as well as BP (Kelley and Kelley, 2009; Cornelissen et al., 2011; Umpierre et al., 2011; Greene et al., 2012; Mann et al., 2014; Álvarez et al., 2019; Amaro-Gahete et al., 2019b). In the present study, BP and the CV risk Z-Score decreased in the concurrent training group, which concurs with the results of other studies involving similar concurrent training interventions (Kelley and Kelley, 2009; Cornelissen et al., 2011; Umpierre et al., 2011; Greene et al., 2012; Mann et al., 2014; Álvarez et al., 2019; Amaro-Gahete et al., 2019b). However, our study findings partially disagree with those previously mentioned (Kelley and Kelley, 2009; Cornelissen et al., 2011; Umpierre et al., 2011; Greene et al., 2012; Mann et al., 2014; Álvarez et al., 2019; Amaro-Gahete et al., 2019b) since we showed no significant differences between the concurrent training group and the control group with respect to the change in the glycemic (i.e., plasma glucose and insulin concentration and HOMA-IR) and lipid (total cholesterol, HDL-C, LDL-C, and TGs) profiles, as well as in hepatic function. The different intervention durations could be a potential reason for these discrepancies (i.e., ranged from 8 to 24 weeks). But certainly, the most plausible explanation is that the low sample size of the present study is not enough to detect statistical differences between groups. Further studies with more statistic power are needed to confirm the current results.

The influence of concurrent training on energy metabolism-related parameters has been previously investigated obtaining controversial findings. On the one hand, a significant increase of RMR was observed after a 10-week concurrent training program in physically active men (Dolezal and Potteiger, 1998), whereas no significant changes were noted neither in RMR nor in resting substrates oxidation in response to both a 20- and 12-week concurrent training interventions in sedentary middle-aged women (Byrne and Wilmore, 2001) and in middle-aged sedentary adults (Amaro-Gahete et al., 2020), respectively. On the other hand, there is also controversy regarding the effects of both aerobic and resistance training on MFO. While no change in MFO was observed after either 4 weeks of aerobic training or 12 weeks of concurrent training in middle-aged adults with (Venables and Jeukendrup, 2008) and without obesity (Amaro-Gahete et al., 2020), a significant increase of MFO was reported in overweight men after a 3-month aerobic training intervention (Rosenkilde et al., 2015), and in middle-aged untrained adults after a 1-year aerobic training intervention (Scharhag-Rosenberger et al., 2010). The present results showed no significant differences in RMR, resting nutrients oxidation, and MFO after 12 weeks of concurrent training compared with a no-exercise control group. These findings could be explained by the lack of changes in LM since this outcome is the most important determinant of RMR (i.e., skeletal muscle is the most metabolically active tissue) (Blundell et al., 2015), and its optimization could improve mitochondrial function/activity and insulin sensitivity modulating in turn substrate oxidation during both resting and exercise conditions (Goodpaster and Sparks, 2017). It is therefore plausible that an increase of LM could be mandatory or determinant to induce changes in energy metabolism-related parameters. In the same line, it is possible that the required exercise duration to guarantee LM and RMR improvements would be longer than 12 weeks.

In the current study, a significant increase of the LV end diastolic diameter was observed following a 12-week concurrent training intervention, which seems logical because this type of exercise training induces subsequent increments of pressure overload to volume overload, as a consequent of the exercise duration and intensity (Hosseini et al., 2012). These findings concurred with those reported by previous studies that revealed LV morphologic adaptations in response to (i) an 8-week concurrent training program in young women (Hosseini et al., 2012), (ii) a 10-week concurrent training intervention in trained men (duManoir et al., 2007), and (iii) a 5-month concurrent training program in rowers (Cavallaro et al., 1993). However, no further changes were observed in other cardiac parameters when both exercise and control groups were compared. This finding could be explained by the relatively short duration of our intervention since previous studies have suggested longer exercise programs to improve cardiac function (Voulgari et al., 2013). Despite the positive changes found only in LV end diastolic diameter, these findings are very relevant due to the particular characteristic of the participants (i.e., obese rather than trained or healthy population) who could especially benefit of the improvement in this morphologic parameter which is known as an indicator of CV health and a risk factor for mortality (Narayanan et al., 2014).

## Limitations

This study had some important limitations that should be noted, and therefore findings of this work should be interpreted with caution. First, the small sample size limits the generalization of the results and might limit the detection of statistical significance. Regardless, the effect size for all outcome measures has been reported. Second, this intervention was conducted in obese men, thus we cannot extrapolate the findings to their women counterparts. Insulin resistance was not assessed by the gold standard method (i.e., the hyperinsulinemic euglycemic glucose clamp technique). However, HOMA (Ascaso et al., 2001) method has been previously validated for assessing insulin resistance. Finally, we observed a reduction of energy intake in the exercise group which could explain the body weight loss of such participants. Nevertheless, it has been suggested in previous studies that the compensatory effect of exercise intervention may be on behavior parameters (e.g., diet and PA) (Stubbs et al., 2004). This phenomenon may have affected our participants but further studies are needed to confirm these findings.

## Conclusion

In conclusion, the present study shows that a 12-week concurrent training intervention is an effective strategy to induce weight and FM loss with simultaneous reductions of BP and cardiometabolic risk, and improving CV function in obese men. These findings could have important clinical implications since, despite its inherent limitations, they suggest that a combination of aerobic and resistance training intervention is an effective and cost-efficient strategy for the management of obesity and its related complications. Further studies should be conducted to confirm these results with a higher sample size, and to determine whether the same holds true for women and whether longer duration would imply additional benefits.

## Data Availability Statement

The raw data supporting the conclusions of this article will be made available by the authors, without undue reservation.

## Ethics Statement

The studies involving human participants were reviewed and approved by the Ethics Committee on Human Research at the University of Leon. The patients/participants provided their written informed consent to participate in this study.

## Author Contributions

JGP-G, JC-P, and DJ-P contributed to conceptualization. FA-G and DJ-P contributed to data curation. FA-G contributed to formal analysis and writing—original draft. DJ-P contributed to funding acquisition, project administration, resources, and supervision. JGP-G, JC-P, DV-D, CL, and DJ-P contributed to investigation. FA-G, JGP-G, JC-P, and DJ-P contributed to methodology. FA-G, JGP-G, JC-P, DV-D, CL, and DJ-P contributed to writing—review and editing. All authors contributed to the article and approved the submitted version.

## Conflict of Interest

The authors declare that the research was conducted in the absence of any commercial or financial relationships that could be construed as a potential conflict of interest.
