# Supplementary figures and images for: Effect of a 12-Week Concurrent Training Intervention on Cardiometabolic Health in Obese Men: A Pilot Study
Source: Front Physiol. 2021 Feb 11;12:630831. doi: 10.3389/fphys.2021.630831 (PMC7905165; doi:10.3389/fphys.2021.630831)

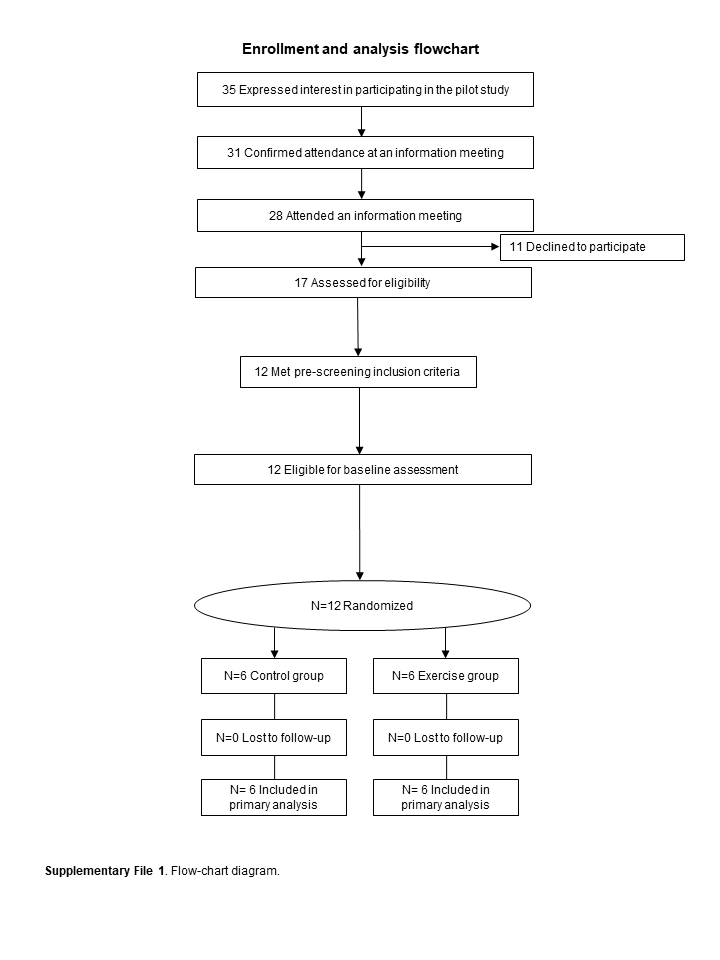

Supplement: Supplementary file 1 [file Image_1.TIF]
